# Brain volumes and white matter microstructure in 8- to 10-year-old children born with fetal growth restriction

**DOI:** 10.1007/s00247-022-05372-0

**Published:** 2022-04-22

**Authors:** Noora Korkalainen, Tero Ilvesmäki, Riitta Parkkola, Marja Perhomaa, Kaarin Mäkikallio

**Affiliations:** 1 Department of Obstetrics and Gynecology, PEDEGO Research Unit, Oulu University Hospital, Aapistie 5 A, 5000, FI-90014 Oulu, PL Finland; 2 University of Oulu, Oulu, Finland; 3 Department of Radiology, Turku University Hospital, Turku, Finland; 4 Department of Radiology, University of Turku, Turku, Finland; 5 Department of Radiology, Oulu University Hospital, Oulu, Finland; 6 Department of Obstetrics and Gynecology, Turku University Hospital, Turku, Finland

**Keywords:** Brain, Children, Diffusion tensor imaging, Fetal growth retardation, Magnetic resonance imaging, Placental insufficiency, Tract-based spatial statistics

## Abstract

**Background:**

Fetal growth restriction caused by placental insufficiency is associated with increased risk of poor neurodevelopment, even in the absence of specific perinatal brain injury. Placental insufficiency leads to chronic hypoxaemia that may alter cerebral tissue organisation and maturation.

**Objective:**

The aim of this study was to assess the effects fetal growth restriction and fetal haemodynamic abnormalities have on brain volumes and white matter microstructure at early school age.

**Materials and methods:**

This study examined 32 children born with fetal growth restriction at 24 to 40 gestational weeks, and 27 gestational age-matched children, who were appropriate for gestational age. All children underwent magnetic resonance imaging (MRI) at the age of 8–10 years. Cerebral volumes were analysed, and tract-based spatial statistics and atlas-based analysis of white matter were performed on 17 children born with fetal growth restriction and 14 children with birth weight appropriate for gestational age.

**Results:**

Children born with fetal growth restriction demonstrated smaller total intracranial volumes compared to children with normal fetal growth, whereas no significant differences in grey or white matter volumes were detected. On atlas-based analysis of white matter, children born with fetal growth restriction demonstrated higher mean and radial diffusivity values in large white matter tracts when compared to children with normal fetal growth.

**Conclusion:**

Children ages 8–10 years old born with fetal growth restriction demonstrated significant changes in white matter microstructure compared to children who were appropriate for gestational age, even though no differences in grey and white matter volumes were detected. Poor fetal growth may impact white matter maturation and lead to neurodevelopmental impairment later in life.

## Introduction

Fetal growth restriction affects 5%–10% of all pregnancies, with the main aetiology being placental insufficiency. Impaired placental blood flow results in chronic exposure to fetal hypoxaemia and malnutrition and may trigger redistribution of blood flow to vital fetal organs, the heart and the brain [1]. The survival of growth-restricted fetuses, especially those born prematurely, has increased significantly in recent years. However, placental and fetal haemodynamic changes influence short- and long-term outcomes, and with regard to neurodevelopmental outcomes, children born with fetal growth restriction show difficulties in neurocognitive processing from infancy into adulthood, even with no specific perinatal brain injury [2–14]. Contradictory to acute hypoxic events, fetal growth restriction with chronic hypoxaemia may cause changes in brain organisation and maturation rather than immediate tissue destruction.

Magnetic resonance imaging (MRI) studies on fetal growth restriction have described structural changes in the brain, especially in children born preterm with impaired fetal growth [15–19]. Smaller total brain and grey matter volumes and delays in cortical development have been reported in children born with fetal growth restriction [15, 17–20]. Diffusion tensor imaging is superior to conventional MRI techniques in detecting white matter microstructural abnormalities [21]. Fractional anisotropy, axial diffusivity, radial diffusivity and mean diffusivity are quantitative water diffusion metrics that allow the exploration of white matter microstructure [22]. This technique has revealed abnormalities in white matter structure, especially in children born prematurely, and according to some human and animal studies, fetal growth restriction contributes to white matter abnormalities [23–29]. However, studies in children and adolescents born with fetal growth restriction report conflicting results [15, 20, 30, 31]. Despite the data accumulated from long-term outcomes of children born with fetal growth restriction, the underlying mechanisms leading to poor neurocognitive development are still widely unclear.

We hypothesised that brain volumes and diffusion parameters of white matter differ between children born with fetal growth restriction and their appropriately grown gestational age-matched peers at the age of 8–10 years. Furthermore, we explored possible associations between fetoplacental haemodynamic findings and brain volumes, as well as white matter diffusion parameters in this cohort of children born with fetal growth restriction and their counterparts with normal fetal growth.

### Patients and methods

**Fig. 1:**
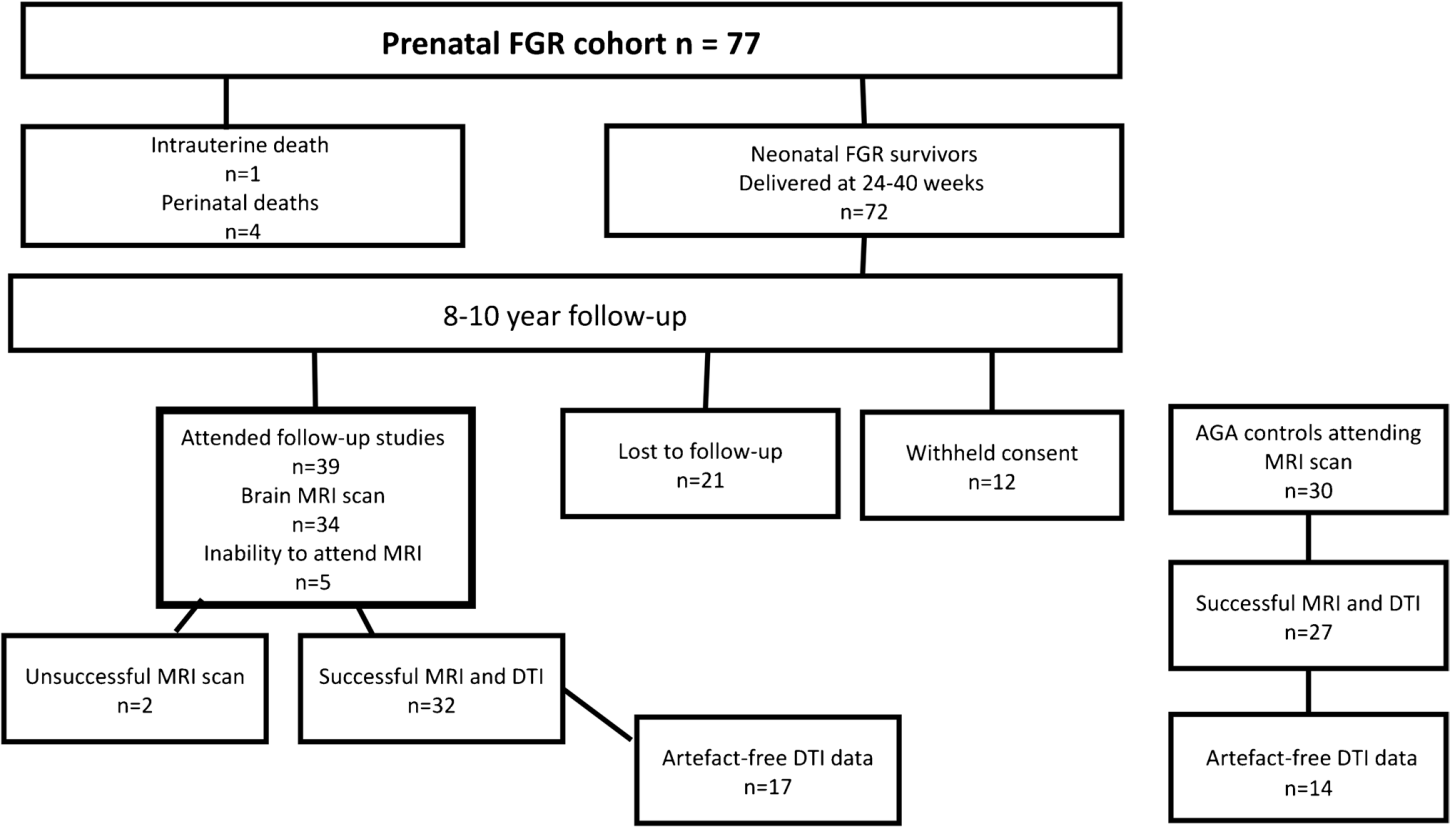
Flowchart of follow-up pathway for 8- to 10-year-old children with fetal growth restriction (FGR). *ABA* atlas-based analysis*, DTI* diffusion tensor imaging, *MRI* magnetic resonance imaging

The participants in this study belonged to a prospectively collected cohort (*n* = 77) of growth-restricted fetuses (birth weight < 10^th^ percentile and/or umbilical artery pulsatility index > 2 standard deviations [SD]) [32, 33]. Mothers were recruited in 1998–2001 from a high-risk prenatal unit, and parents were subsequently contacted to book a follow-up visit for their children at 8–10 years of age. In all cases, gestational age was confirmed by ultrasound before 20 gestational weeks. Pregnancies with major structural and chromosomal abnormalities and those complicated by chorioamnionitis and/or ruptured membranes were excluded. From the original cohort, 32 children underwent an MRI of the head. The control group consisted of 27 children with growth appropriate for gestational age (AGA, birth weight > 10^th^ percentile). The control group was selected from delivery room records and matched for gestational age, gender, mode of delivery and delivery within ± 2 weeks of the index neonate born with fetal growth restriction. Eleven controls and eight children born with fetal growth restriction were excluded from the diffusion tensor imaging analyses due to eddy current artefacts, which compromised the diffusion tensor imaging tensor fitting process (Fig. 1). The research protocol was approved by the Ethical Committee of Oulu University Hospital (approval number 8/2008 on Feb. 21, 2008). Study participation required written parental consent.

### Prenatal assessment

Maternal characteristics and obstetric data were collected at study entry. Maternal hypertensive disorders were categorised according to the guidelines of the American College of Obstetrics and Gynecology [34] and prenatal steroid therapy included two 12-mg betamethasone doses 24 h apart.

Detailed information concerning placental and fetal haemodynamic assessments collected by a single investigator, K.M., (a specialist in maternal–fetal medicine with 24 years of experience) within a week (median: 2.9 days) before delivery has been described previously [35]. Sequoia 512 (Acuson, Mountain View, CA) with 4- to 8-MHz transducers were used for scanning and the angle of insonation was maintained at < 15 degrees in all examinations. Three consecutive cardiac cycles were obtained, and the mean values were used in the analyses. The pulsatility index (PI) in the umbilical artery (UA) was measured from the free loop of the umbilical cord and further categorised as normal, increased (UA PI > 2 SD) or absent/reversed end-diastolic flow [33, 36]. The middle cerebral artery (MCA) pulsatility index was assessed, and the cerebroplacental ratio (CPR) was calculated as the MCA PI/UA PI [37]. Significant placental insufficiency (UA PI > 2 SD) and redistribution of blood flow (CPR < ^−^2 SD) were determined according to earlier published data [38, 39].

The managing obstetricians were blinded to the study data. From 1998 to 2001, the indications for delivery were 1) worsening maternal condition, 2) abnormal non-stress test in fetal heart rate monitoring and 3) abnormal pulsatility in the ductus venosus. Decisions to deliver were made by senior consultants with more than 5 years of experience in the labour ward.

### Postnatal outcomes

  At birth, the mode of delivery, umbilical artery blood gas values, Apgar scores and neonatal morphometric measurements were recorded. All children born before 32 gestational weeks underwent ultrasound imaging of the brain in the neonatal period. Brain ultrasound and/or MRI imaging was performed for infants born after 32 weeks when clinically indicated. Children attended follow-up studies at a mean age of 9.1 years (Table 1). The children’s medical charts were reviewed for diagnoses.

**Table 1:** Perinatal and postnatal characteristics of children born with fetal growth restriction (FGR) and children who were appropriate for gestational age (AGA). The values given are mean (± standard deviation [SD]), median (range) and *n* (%)

	FGR (*n* = 32)	AGA (*n* = 27)	*P*^a^
Maternal			
Age at delivery (mean, years)	30 (5)	31 (6)	0.47
Hypertensive disorder or preeclampsia	13/32(40%)	1/27 (4%)	**0.001**^b^
Education			
University	17/32 (53%)	13/26 (50%)	1.00
Vocational/basic education	15/32 (47%)	13/26 (50%)	
Fetal			
Umbilical artery ARED	6/32 (19%)	0	
Umbilical artery PI > 2 SD	22/32 (69%)	0	
Middle cerebral artery PI < -2 SD	10/32 (31%)	0	
CPR -2 SD	16/32 (50%)	0	
Delivery			
Gestational age (mean, weeks)	34 (4)	35 (4)	0.65
Caesarean delivery	21/32 (65%)	21/26 (81%)	0.25
Birth weight (mean, grams)	1736 (711)	2683 (1025)	**0.004**^c^
Birth weight percentile (mean)	4.9 (8.4)	62.4 (21.7)	** < 0.001**^c^
Male	15/32 (47%)	12/27 (44%)	1.00
1-min Apgar (mean)	8 (2)	8 (2)	0.14
5-min Apgar (mean)	8 (1)	8 (1)	0.89
Umbilical artery pH (mean)	7.28 (0.06)	7.26 (0.05)	0.67
Postnatal			
NICU (median, days)	0 (0–116)	0 (0–71)	1.00
Mechanical ventilation	11/31 (36%)	8/23 (35%)	1.00
Respiratory distress syndrome	8/31 (26%)	8/27 (30%)	0.78
Bronchopulmonary dysplasia	4/31 (13%)	1/27 (4%)	0.36
Intraventricular haemorrhage^d^	0	2/27 (7%)	0.21
Periventricular leukomalacia^d^	0	0	
Necrotizing enterocolitis	0	0	
Retinopathy of prematurity	1/31 (3%)	2/27 (7%)	0.59
Sepsis	5/30 (17%)	1/23 (4%)	0.22
Early school age			
Age at MRI scan (mean, years)	9.2 (0.4)	9.1 (0.3)	0.38
Weight (mean, kilograms)	29.1 (5.7)	31.0 (6.8)	**0.048**^c^
Height (mean, centimetres)	132.0 (6.4)	134.9 (7.1)	0.17
Head circumference (mean, centimetres)	52.5 (2.0)	53.4 (1.0)	**0.048**^c^
Education			
Mainstream education	25/32 (78%)	26/27 (96%)	0.06
Modified education	7/32 (22%)	1/27 (4%)	

*ARED* absent or reversed end-diastolic velocity, CPR cerebroplacental ratio, *NICU* neonatal intensive care unit, *PI* pulsatility index

^a^ *P*-value < 0.05 is significant (bold)

^b^ Fisher exact test

^c^ Student’s *t*-test

^d^ all children born at gestational age < 32 weeks underwent brain ultrasound in the neonatal period, and children born after 32 gestational weeks underwent brain imaging when clinically indicated

### Magnetic resonance imaging and data analysis

An 8-channel head coil was used for brain MRI. The child’s head was surrounded by soft cushions during scanning, and ear plugs were used to protect the child from imaging noise. The children received no sedation. Conventional MRI was performed using a 1.5-T Signa HDX scanner (GE Healthcare, Milwaukee, WI). The study protocol comprised a T1-weighted sagittal spin echo sequence. For this protocol, the slice thickness was 5 mm with a 1-mm gap between slices, the field of view was 24 cm with a 512 × 512 matrix, and the repetition time (TR)/echo time (TE) was 540/14 ms. In addition, T2-weighted axial images were taken using the PROPELLER (Periodically Rotated Overlapping ParallEL Lines with Enhanced Reconstruction) technique with a slice thickness of 5 mm and a 1-mm gap, an echo train length of 28, a reconstruction diameter of 22 cm with a 512 × 512 matrix and a TR/TE of 5,000/173 ms.

Diffusion tensor imaging was obtained using a spin echo echoplanar sequence with 3-mm isotropic voxels, 40 gradient directions and a b value of 1,000. The TR was 9,000 ms and the TE was as short as possible. The slice thickness was 3 mm and the field of view was 192 mm with a 64 × 64 matrix. Two radiologists, M.P. and R.P. with 7 and 28 years of experience in paediatric radiology, respectively, analysed the MRI scans.

### Statistical analyses

Statistical analyses of demographic parameters and volumetric imaging data were performed using SPSS 27.0 (IBM, Armonk, NY). Categorical perinatal and postnatal data were compared with chi-square analysis and the Fisher exact test. For continuous variables, the Student’s *t*-test was employed if the data were normally distributed, otherwise the Mann–Whitney *U* test was chosen. A two-tailed *P*-value < 0.05 was selected as the level of statistical significance.

In addition to volumetric analysis, voxel-based morphometry was used to study possible differences in grey matter volumes [40]. The statistical analysis of grey matter volume was performed in its entirety with SPM12 software (https://www.fil.ion.ucl.ac.uk/spm/), using the CAT12 (Computational Anatomy Toolbox v.12.5, http://www.neuro.uni-jena.de/cat/) extension with default settings provided by the CAT12 analysis pipeline. The T1-weighted image data were first converted to the Neuroimaging Informatics Technology Initiative (Nifti) format, normalized to the 1.5-mm Montreal Neurological Institute (MNI) space and segmented to grey and white matter and corticospinal fluid using Diffeomorphic Anatomical Registration Through Exponentiated Lie Algebra (DARTEL) registration [41]. A Gaussian smoothing kernel with full width at half maximum of nine voxels was applied to the normalized data. Voxel-based morphometry analysis was conducted between the children born with fetal growth restriction (*n* = 32) and the control children appropriate for gestational age (*n* = 27), and between the subgroup of children born with fetal growth restriction and abnormal umbilical artery blood flow (UA PI > 2 SD) (*n* = 22) and the children born appropriate for gestational age. A two-sample *t*-test with gestational age and total intracranial volume as covariates was used in the voxel-based morphometry analysis. Only family-wise error-corrected *P*-values under 0.01 were considered statistically significant.

Whole-brain voxel-wise statistical analysis of the diffusion tensor imaging data was carried out using tract-based spatial statistics [42] a part of the Functional MRI of the Brain Software Library (FSL) version 6.0 (https://fsl.fmrib.ox.ac.uk/fsl/fslwiki) [43]. Diffusion tensor imaging parameter fitting and pre-processing were performed using built-in tools of the FSL package. The diffusion data were corrected for susceptibility distortions, eddy currents, inter- and intra-volume subject movement and signal dropout using the inbuilt eddy tool [44, 45]. The eddy tool can remedy even some of the graver artefacts in diffusion data using nonparametric mathematical correction algorithms [46]. After preprocessing the data, a mean fractional anisotropy image was derived and thinned to create a mean fractional anisotropy skeleton. The threshold for fractional anisotropy values for the creation of the skeleton was chosen as ≥ 0.22. The subjects were registered to study specific target instead of the adult standard-space fractional anisotropy image. Each subject’s aligned data were then projected onto this skeleton for each diffusion tensor imaging parameter, and the resulting skeletonised data were fed into a voxel-wise cross-subject analysis.

Statistical whole-brain group comparisons were performed using tract-based spatial statistics and randomized with threshold-free cluster enhancement [47]. A nonparametric two-sample *t*-test with a general linear model design was used for statistics [48]. Inference was obtained through 15,000 permutations, testing the resulting clusters for significance at *P* ≤ 0.05 (one-sided), corrected for multiple comparisons across space.

  A tract-based spatial statistics group comparison between the group of children born with fetal growth restriction and the control group of children who were appropriate for gestational age was performed using the artefact-free diffusion tensor imaging data (17 fetal growth restriction, 14 appropriate for gestational age) (Table 2). A subgroup analysis was also conducted to study whether the effects of abnormal umbilical artery blood flow (UA PI > 2 SD) (*n* = 8) can be observed via diffusion imaging at the age of 8–10 years. The analyses were adjusted for the effects of gestational age by defining it as a confounding factor in the general linear model used.

**Table 2:** Perinatal and postnatal characteristics of diffusion tensor imaged children born either with fetal growth restriction (FGR) or appropriate growth for gestational age (AGA). The values given are mean (± standard deviation [SD)]), median (range) and *n* (%)

	FGR (*n* = 17)	AGA (*n* = 14)	*P*^a^
Maternal			
Age at delivery (mean, years)	28 (6)	33 (6)	**0.024**^b^
Hypertensive disorder or preeclampsia	6/17 (35%)	0/14(0%)	**0.024**^c^
Education			
University	8/17 (47%)	7/17 (41%)	1.00
Vocational/basic education	9/17 (53%)	7/14 (50%)	
Fetal			
Umbilical artery ARED	2/17 (12)	0	
Umbilical artery PI > 2 SD	8/17 (47)	0	
Middle cerebral artery PI -2 SD	3/12 (25%)	0	
CPR < -2 SD	4/12 (33%)	0	
Delivery			
Gestational age (median, weeks)	37 (24–40)	35 (27–39)	0.25
Caesarean delivery	9/17 (53%)	10/14 (71%)	0.46
Birth weight (mean, grams)	1970 (751)	2649 (1200)	0.064
Birth weight percentile (median)	4.0 (0.1–41.6)	71.3 (37.6–96.9)	** < 0.001**^d^
Male	8/17 (47%)	5/14 (36%)	0.72
1-min Apgar (mean)	7 (3)	8 (1)	0.17
5-min Apgar (mean)	8 (2)	9 (1)	0.73
Umbilical artery pH (mean)	7.30 (0.06)	7.29 (0.06)	0.67
Postnatal			
NICU (median, days)	0 (0–116)	0 (0–71)	0.87
Mechanical ventilation	4/17 (24%)	3/12 (20%)	1.00
Respiratory distress syndrome	4/17 (24%)	3/14 (21%)	1.00
Bronchopulmonary dysplasia	3/17 (18%)	1/13 (8%)	0.62
Intraventricular haemorrhage	0	0	
Necrotizing enterocolitis	0	0	
Retinopathy of prematurity	1/17 (6%)	1/13 (8%)	1.00
Sepsis	2/17 (12%)	1/12 (8%)	1.00
Early school age			
Age at MRI scan (mean, years)	9.1 (0.3)	9.1 (0.3)	0.71
Weight (mean, kilograms)	27.7 (4.9)	33.1 (8.4)	0.08
Height (mean, centimetres)	132.3 (6.7)	137.9 (7.5)	0.08
Head circumference (mean, centimetres)	52.4 (2.1)	53.4 (1.2)	0.17
Education			
Mainstream education	15/17 (88%)	13/14 (93%)	1.00
Modified education	2/17 (12%)	1/14 (7%)	

*ARED* absent or reversed end-diastolic velocity, *CPR* cerebroplacental ratio, *MRI* magnetic resonance imaging, *NICU* neonatal intensive care unit, *PI* pulsatility index

^a^ *P*-value < 0.05 is significant (bold)

^b^ Student’s *t*-test

^c^ Fisher exact test

^d^ Mann–Whitney *U* test

To complement the whole-brain tract-based spatial statistics analyses, atlas-based analyses were performed using regions of interest (ROI) taken from the JHU ICBM-DTI-81 (Johns Hopkins University, International Consortium for Brain Mapping) white-matter probabilistic tractography atlas [49] included in the FSL package. The procedure for the atlas-based analysis was as follows: (i) the ICBM fractional anisotropy map was linearly registered to each of the subjects’ fractional anisotropy map, saving the linear transform matrices; (ii) the linearly transformed fractional anisotropy maps were then nonlinearly registered to the corresponding subject’s fractional anisotropy map, and the warp matrices were again saved; (iii) the saved linear and nonlinear transformations were then applied to the JHU probabilistic tractography atlas, yielding tractography ROIs in each of the subjects’ native space; (iv) the registered ROIs were cropped to include voxels with a minimum of 20% probability and were then used to obtain weighted means for the corresponding tractographic regions, and (v) the obtained regional weighted means were fed into the statistical program (JASP Team 2020. JASP Version 0.14.1) for analysis. The statistical analyses were performed using analysis of covariance (ANCOVA), while controlling for gestational age. Both uncorrected (*P* ≤ 0.05) and corrected (*P* ≤ 0.01) results are presented in the results.

## Results

The perinatal and postnatal clinical characteristics of the study population are shown in Tables 1 and 2. As expected, in the group of children born with fetal growth restriction, maternal hypertensive disorders were more common and birth weight and birth weight percentiles were lower than in the group of children born appropriate for gestational age. No differences in the UA acid base status and 1- and 5-min Apgar scores were detected between the groups and neither did the short-term outcomes including brain imaging during the perinatal period differ. During the follow-up visit at a mean age of 9 years, the mean (SD) weight and height of the children born with fetal growth restriction were 29.1 (5.7) kg and 132 (6) cm, respectively, and the respective measures were 31.0 (6.8) kg and 135 (7) cm in the group of children born appropriate for gestational age (*P* = 0.048). The mean (SD) head circumferences in the group of children born with fetal growth restriction and control groups were 52.5 (2.0) cm and 53.4 (1.0) cm, respectively (*P* = 0.048). There were no significant differences in the age or in gender proportion of the compared groups at the time of the follow-up investigations. Except for one previously undiagnosed Arnold Chiari malformation type I in a child born appropriate for gestational age with a normal neurodevelopmental outcome, no other clinically significant brain pathologies were detected on MRI.

The children born with fetal growth restriction demonstrated significantly smaller total intracranial volumes than the children born appropriate for gestational age (Table 3). No statistically significant differences in grey or white matter volumes or the amount of cerebrospinal fluid were detected between the compared groups (Table 3). Similar findings were made in the subgroup analysis between children born with fetal growth restriction and abnormal umbilical artery blood flow (UA PI > 2 SD; n = 22) and control children born appropriate for gestational age. No significant differences in brain volumes were found in the comparisons between children born with fetal growth restriction presenting cerebral redistribution prenatally (< -2 SD; *n* = 16) and control children born appropriate for gestational age (Table 3), and neither did we find any significant differences in grey matter regional volumes evaluated by voxel-based morphometry between compared groups, not even in the comparisons between children born with fetal growth restriction and abnormal umbilical artery blood flow (UA PI > 2 SD) and children born appropriate for gestational age (data not shown).

**Table 3:** Cerebral tissue volumes (cm^3^) at the age of 8–10 years in children born with fetal growth restriction (FGR), FGR children with prenatally detected significant placental insufficiency (umbilical artery pulsatility index [UA PI] > 2 standard deviations [SD]), FGR children with prenatal cerebral redistribution (cerebroplacental ratio [CPR] < -2 SD) and in children with appropriate growth for gestational age (AGA). The values given are mean (± standard deviation [SD])

Volume (cm^3^ [SD])	FGR (*n* = 32)	FGR UA PI > 2 SD (*n* = 22)	FGR CPR < -2 SD (*n* = 16)	AGA (*n* = 27)
Total intracranial volume	1,382^a^	1,375^b^	1,384	1,452
	(152)	(130)	(155)	(93)
Grey matter	746	741	744	770
	(60)	(54)	(60)	(51)
White matter	427	425	422	442
	(52)	(44)	(51)	(43)
Cerebrospinal fluid	209	208	218	240
	(64)	(64)	(72)	(61)

^a^ *P* < 0.05 FGR vs. AGA

^*b*^* P* < *0.05* FGR UA PI > 2 SD vs. AGA

Data analysed by Student’s *t*-test

**Fig. 2:**
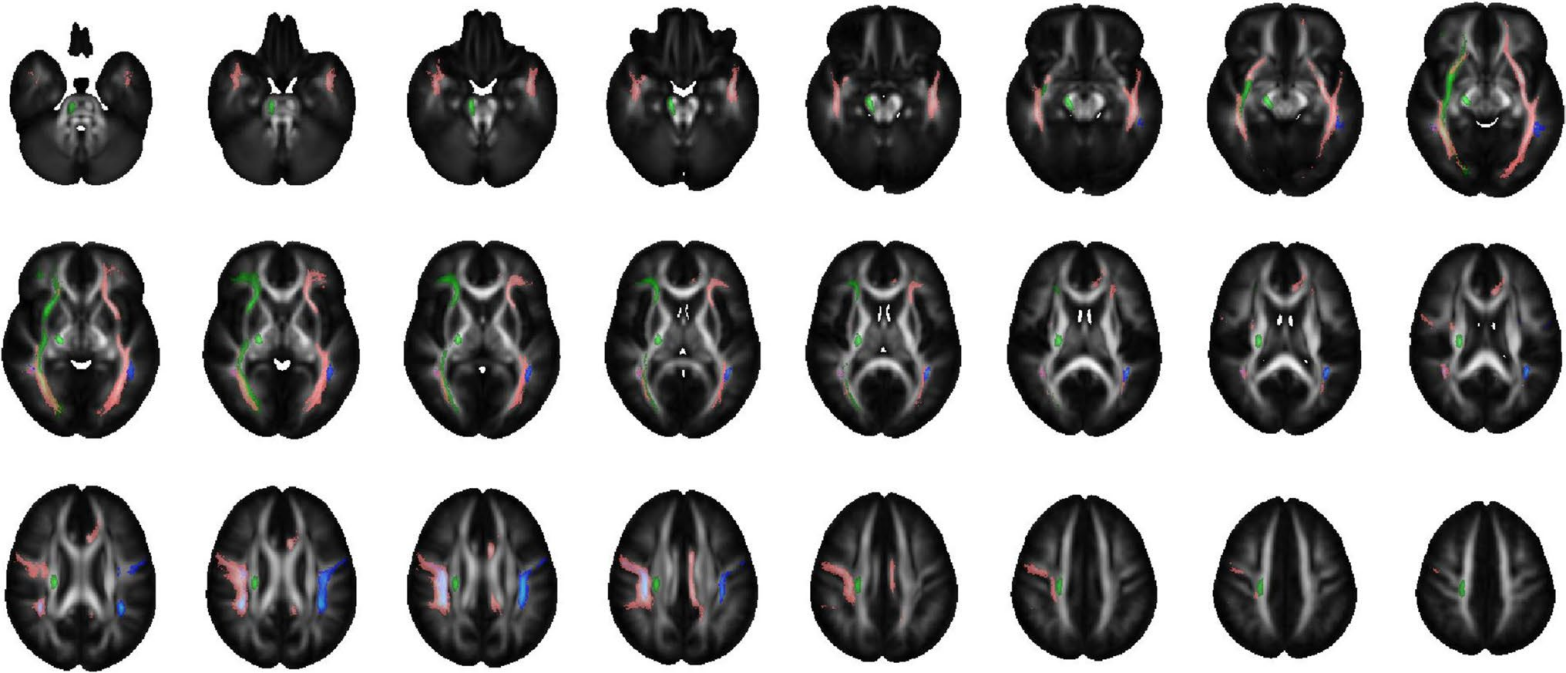
Atlas-based analysis. Transverse slices of the Johns Hopkins University, International Consortium for Brain Mapping fractional anisotropy map with overlays of the atlas regions show significant (*P* < 0.05) differences between children born with fetal growth restriction and children appropriate for gestational age. Regions with differences in mean diffusivity, radial diffusivity and multiple diffusion tensor imaging metrics are depicted in the figure as blue, green and pink, respectively. Radiologic convention (left = right)

In the tract-based spatial statistics analysis of 17 children born with fetal growth restriction and 14 children who were appropriate for gestational age, no diffusion tensor imaging metric differences were detected, not even in the comparisons between children born with fetal growth restriction and abnormal umbilical artery blood flow (UA PI > 2 SD) and the control group (data not shown). In exploring the cerebral white matter with atlas-based analysis, the group of children born with fetal growth restriction showed several tract areas with significantly (*P* < 0.05) lower fractional anisotropy values and higher mean diffusivity, radial diffusivity and axial diffusivity values than the group of children born appropriate for gestational age (Table 4, Fig. 2). The subgroup analysis of children born with fetal growth restriction and abnormal umbilical artery blood flow (UA PI > 2 SD) revealed higher mean diffusivity values in the left cingulate gyrus than values observed in the control group (Table 4). However, applying family-wise error correction (*P* < 0.01) restricted the results to the main group analysis, where higher mean diffusivity and radial diffusivity were found in the left cingulate gyrus, left inferior fronto-occipital fasciculus and left inferior longitudinal fasciculus (Table 4). Higher mean diffusivity was also found in the right inferior longitudinal fasciculus.

**Table 4:** Detailed atlas-based analysis results of the tract regions with significant differences between children born with fetal growth restriction (FGR) and appropriate for gestational age (AGA) evaluated by diffusion tensor imagining (DTI) at the age of 8–10 years. Metrics were compared by analysis of covariance (ANCOVA) with gestational age as a covariate. Fractional anisotropy (FA) is unitless, and axial diffusivity (AD), mean diffusivity (MD) and radial diffusivity (RD) are expressed as 10^–3^ mm^2^/s. The values given are mean (standard deviation)

Region of interest	DTI metric mean (SD)	FGR (*n* = 17)	AGA (*n* = 14)	*P*-value (ANCOVA)
Cingulum (cingulate gyrus) L	MD	0.827 (0.017)	0.805 (0.015)	0.001^a^
Cingulum (cingulate gyrus) L	RD	0.651 (0.016)	0.629 (0.022)	0.002^a^
Corticospinal tract R	RD	0.570 (0.022)	0.552 (0.031)	0.046^b^
Inferior fronto-occipital fasciculus L	MD	0.847 (0.015)	0.830 (0.018)	0.006^a^
Inferior fronto-occipital fasciculus L	RD	0.659 (0.018)	0.638 (0.024)	0.008^a^
Inferior fronto-occipital fasciculus R	RD	0.667 (0.029)	0.648 (0.027)	0.046^b^
Inferior longitudinal fasciculus L	FA	0.359 (0.014)	0.374 (0.022)	0.020^b^
Inferior longitudinal fasciculus L	MD	0.858 (0.020)	0.837 (0.019)	0.009^a^
Inferior longitudinal fasciculus L	RD	0.683 (0.019)	0.659 (0.023)	0.003^a^
Inferior longitudinal fasciculus R	MD	0.859 (0.029)	0.834 (0.027)	0.009^a^
Inferior longitudinal fasciculus R	RD	0.669 (0.028)	0.644 (0.030)	0.017^b^
Superior longitudinal fasciculus (temporal part) L	MD	0.807 (0.020)	0.788 (0.024)	0.038^b^
Superior longitudinal fasciculus (temporal part) R	MD	0.800 (0.027)	0.777 (0.024)	0.030^b^
Superior longitudinal fasciculus R	MD	0.811 (0.021)	0.789 (0.021)	0.013^b^
Superior longitudinal fasciculus R	RD	0.646 (0.027)	0.624 (0.026)	0.050^b^
Superior longitudinal fasciculus R	AD	1.142 (0.023)	1.120 (0.021)	0.015^b^
Uncinate fasciculus R	FA	0.339 (0.032)	0.370 (0.027)	0.012^b^
Uncinate fasciculus R	RD	0.697 (0.026)	0.675 (0.025)	0.025^b^
Region of interest	DTI metric	FGR with UA PI > 2 SD (*n* = 8)	AGA (*n* = 14)	*P*-value
Cingulum (cingulate gyrus) L	MD	0.819 (0.012)	0.805 (0.015)	0.033^b^

*L* left, *R* right, *UA PI* umbilical artery pulsatility index

^a^ Significant at a level of *P* < 0.01 (family-wise error corrected)

^b^ Significant at a level of *P* < 0.05

## Discussion

In this study, 8- to 10-year-old children born with fetal growth restriction at 24–40 gestational weeks had smaller total intracranial volumes than their peers with normal fetal growth, with no difference in grey and white matter volumes. Neither did grey and white matter volumes differ between children born appropriate for gestational age and the subgroup of children born with fetal growth restriction presenting significant placental insufficiency (UA PI > 2 SD) prenatally. Tract-based spatial statistical evaluation of diffusion tensor imaging measures did not show any differences between the children born with fetal growth restriction and children born appropriate for gestational age, but the more detailed atlas-based analysis revealed differences in white matter tracts between the compared groups, suggesting differences in white matter maturation at early school age.

Clinical imaging biomarkers would be of great value to aid with the identification of individuals born with fetal growth restriction and at increased risk for abnormal neurodevelopment. Cerebral tissue volumes and diffusion tensor imaging might reveal clinically important information and help plan therapeutic measures for neonates with a high risk of adverse neurodevelopment. However, interpretation of various study results is difficult due to differences in the studied fetal growth restriction phenotypes, age at evaluation and methods used.

In the present study, children born with fetal growth restriction showed smaller head circumferences and total intracranial volumes than children born appropriate for gestational age, but no differences were found in grey or white matter volumes or regional grey matter volumes analysed by voxel-based morphometry between the compared groups at the age of 8–10 years. Previously, smaller grey matter volumes have been reported in children born with fetal growth restriction at 24–34 weeks and examined at 0–1 year of corrected age in cohorts of 14–20 children [15, 18, 31]. Tolsa et al. [15] found significantly reduced intracranial and grey matter volumes in a cohort of 14 infants born with severe fetal growth restriction and abnormal umbilical artery blood flow, delivered at 30–34 weeks’ gestational age and compared with appropriately grown infants of similar gestational ages. Similarly, in a cohort of 20 children with severe fetal growth restriction and abnormal umbilical artery blood flow (UA PI > 95^th^ percentile) delivered at 26–34 weeks and examined at the corrected age of 1 year, children born with fetal growth restriction showed smaller total brain volumes and grey matter volumes compared to both preterm and term children born with appropriate fetal growth [31]. Interestingly, head circumference differences were detected only between children born with fetal growth restriction and preterm children with appropriate fetal growth, but not between children born with fetal growth restriction and term born controls with appropriate fetal growth [31]. Neither of these studies showed differences in white matter volumes between children born with fetal growth restriction and children born appropriate for gestational age [15, 31]. In the study by Tolsa et al. [15], the difference between grey matter volumes in children born with fetal growth restriction and abnormal umbilical artery blood flow profiles and children born appropriate for gestational age reduced by 25% in the time interval from birth to the corrected age of 0 (40 weeks of pregnancy). Our cohort, as well as the cohort of 23 preterm born children with fetal growth restriction and severely abnormal umbilical artery blood flow examined by Morsing et al. [20] at the age of 7–8 years, showed no differences in grey or white matter volumes compared to preterm and term children with appropriate fetal growth. Morsing et al. concluded that brain volumes at early school age in severe fetal growth restriction were primarily associated with the degree of prematurity at birth and less with poor fetal growth [20]. Our cohort included children with early-onset and late-onset fetal growth restriction, and we speculate that postnatal maturation, catch-up growth and other factors have an impact on cerebral findings at 8–10 years. It is worth noting that, unlike some other studies, our cohort did not include children whose delivery was complicated by chorioamnionitis or who had birth asphyxia. In addition, the fraction of children who needed mechanical ventilation during the perinatal period was moderate in our study. In volumetric and diffusion tensor imaging analysis, 36% and 24% of the children born with fetal growth restriction, and 35% and 20% of children born appropriate for gestational age, were mechanically ventilated in the neonatal period. This is in line with the results of the TRUFFLE trial, performed in 2005–2010 [50]. The rate of mechanical ventilation may naturally impact the MRI results. However, to avoid the bias caused by the clinical care, the children born with fetal growth restriction and controls with normal fetal growth were from the same time-period to ensure that their clinical treatment protocols did not differ.

Contrary to acute adverse events antenatally and during labour, fetal growth restriction is a chronic condition that might induce reorganisation of cerebral tissue and impact maturation of the brain rather than cause tissue destruction [51]. Therefore, in addition to detecting major complications in the brain, applied MRI modalities have been employed to identify subtle cerebral changes. Diffusion tensor imaging parameters seem to correlate well with cerebral maturation and organisation in fetal, as well as postnatal, life [23, 24]. Therefore, we were interested in exploring whether diffusion tensor imaging metrics differ between children born with fetal growth restriction and children born appropriate for gestational age at early school age.

Our tract-based spatial statistics findings revealed no differences in diffusion parameters between children born with fetal growth restriction (including those with abnormal umbilical artery blood flow) and children born appropriate for gestational age. In the atlas-based analysis results, the group of children born with fetal growth restriction showed lower fractional anisotropy and higher mean diffusivity, radial diffusivity and axial diffusivity values than the control group, and in nine children born with fetal growth restriction and abnormal umbilical artery blood flow, the mean diffusivity values were higher than in the control group. Our findings are in line with studies by Lepomäki et al. [28] and Eixarch et al. [52], who reported decreased fractional anisotropy in several areas in preterm and term-born children with fetal growth restriction, examined between a corrected age of 0–1 years. Padilla et al. [31] found decreased fractional anisotropy in the corpus callosum and increased fractional anisotropy in several tracts in children born with fetal growth restriction examined at the age of 1 year compared to preterm ad term children with appropriate fetal growth. Saunavaara et al. [29] reported lower fractional anisotropy and higher radial diffusivity in several white matter areas at early school-age in preterm children born with fetal growth restriction compared to term children with normal fetal growth, whereas no differences were found between preterm born children with fetal growth restriction and preterm children with normal fetal growth. Similarly, in young adolescence, 46 children born small for gestational age demonstrated lower fractional anisotropy than those with appropriate fetal growth [30]. During cerebral maturation, the structural arrangement and permeability of diffusion barriers in white matter change, but the precise causes of diffusion tensor imaging metric changes are unknown, although a relationship to myelination, diameter, organisation and packing of axons has been suggested [53]. There is consensus that diffusion tensor imaging metrics change with advancing age from the fetal period into adulthood [54, 55] and changes are steepest before early school age, plateauing in late childhood. In general, fractional anisotropy increases and radial diffusivity and mean diffusivity decrease as the brain matures [56]. Previous studies demonstrate that variability in diffusion parameters in young children can be marked even in younger age groups, but the most significant changes in diffusion parameters occur with advancing age when cerebral tissues mature [57]. Since variation in the age of the studied children was small (only 15 months) and there were no significant differences in the actual and corrected ages of the studied groups at the time of MRI scans (accounting for gestational age at delivery), we consider that the effect of age was minimised in our study setting.

In the present cohort, none of the children studied at the age of 8–10 years had clinical signs of cerebral abnormalities during the neonatal period, and they were born with neither asphyxia nor chorioamnionitis, indicating that the studied cohort represents a group of children born with fetal growth restriction with excellent short-term outcomes, as their neonatal characteristics show. Further, differences in anthropometric parameters between the children born with fetal growth restriction and children born appropriate for gestational age at the age of 8–10 years were significant but clinically small, implying good catch-up growth during the early years in children born with fetal growth restriction. Children born with fetal growth restriction who presented artefact-free diffusion tensor imaging data demonstrated lower weight and height compared to the controls, but no differences were found in their head circumferences at 8–10 years of age. It has been speculated that early perinatal and neonatal injuries with haemorrhage play a significant role in the recuperation of cerebral tissue and long-term outcomes [58]. We speculate that these issues have an impact on the negative findings of the tract-based spatial statistics analyses in this cohort.

Our atlas-based analysis demonstrated that children born with fetal growth restriction had increased mean diffusivity and radial diffusivity values in several large white matter tracts, including cingulum, corticospinal tract, inferior longitudinal fasciculus, superior longitudinal fasciculus and uncinate fasciculus compared to children born with appropriate growth. In addition, children born with fetal growth restriction demonstrated decreased fractional anisotropy values in the inferior longitudinal fasciculus and uncinate fasciculus. Similar findings in tract-based spatial statistics analyses have been reported in comparisons between preterm children born with fetal growth restriction and neonates with normal fetal growth [28] as well as preterm children born with fetal growth restriction compared to term children with normal fetal growth at the age of 9 years [29]. Hence, in these studies, the differences between the compared groups may have been more robust and thus visible also in tract-based spatial statistics, while we compared children born with fetal growth restriction at 24–40 weeks with gestational-age matched children with normal fetal growth to examine the effect of impaired fetal growth independently of gestational age. In our cohort, the children born with fetal growth restriction and successful diffusion tensor imaging were delivered at a later mean gestational age of 37 weeks and in good condition with a low incidence of major perinatal complications, and these children also showed good catch-up growth of the head. Furthermore, according to our previous study on this cohort of children born with fetal growth restriction [4], only two of the children evaluated by tract-based spatial statistics and atlas-based analysis demonstrated abnormal neurocognitive development determined as developmental disability or developmental delay requiring continuous therapy. Despite these clinical facts, atlas-based analysis revealed significant differences in several white matter tracts, suggesting that fetal growth impacts white matter maturation and may have later clinical implications. In preterm born children evaluated at 7 years of age, similar to our findings, changes in the superior longitudinal fasciculus, cingulum and corticospinal tract associated significantly with neurodevelopmental impairment [59].

Fetal growth restriction with chronic hypoxaemia may disturb brain maturation and subject these individuals to later neurocognitive difficulties. Given that cerebral maturation, myelination and axonal growth in the white matter mainly take place in the latter half of pregnancy, prematurity may have a significant impact on later neurocognitive outcome [60]. The prefrontal cortex and frontostriatal tracts are part of a wide neural network and convey cognitive processes [61]. During fetal development, the frontal cortex has the longest maturational window, with premature delivery potentially impacting its development significantly. In our study, the fetuses were delivered at 24–40 gestational weeks, whereas previous studies have predominantly included subjects born before 34 weeks. Prematurity may affect long-term neurodevelopment and has been associated with changes in white matter microstructure [25–27]. To specifically evaluate the effect of fetal growth restriction on brain volumes and white matter microstructure, we compared children born with fetal growth restriction to gestational age-matched children with normal fetal growth, resulting in preterm and term born children in both groups. Since there were no significant differences in grey and white matter volumes and we were unable to detect significant differences in tract-based spatial statistics analysis, we agree with Morsing et al. [20], who speculated that gestational age at delivery has a crucial impact on later neurocognitive outcome. Significant differences in white matter microstructure in atlas-based analysis of large white matter tracts between children born with fetal growth restriction and children with normal fetal growth suggest that fetal growth restriction has a significant effect on brain maturation, and hence may also affect neurodevelopmental outcome in these children. In our study, tract-based spatial statistics and atlas-based analysis revealed differing results. In our atlas-based analysis, the atlas of the adult brain was used as a reference, and thus the method was not as optimal as it could have been if using a paediatric atlas. Tract-based spatial statistics, however, is a widely used, well-established mathematical voxel-based method, although it also has shortcomings [62]. Previous reports have widely used tract-based spatial statistics in their analyses [28, 29, 31]. We speculate that the differences between our tract-based spatial statistics and atlas-based analysis and previous findings reported in the literature may partly be a consequence of the ROI method’s higher statistical power when compared to voxel-wise methods [63]. In a previous study by Wen et al. [64], the atlas-based analysis approach revealed results in each of the studied diffusion metrics, whereas tract-based spatial statistics was able to distinguish differences in only half of the studied metrics. The various registration steps in tract-based spatial statistics analysis cause significant warping of the image data, which may also bias results. Our atlas-based analysis did not have the same problem, since subjects’ images were not warped in the analysis, and the atlas-based analysis findings in the present study may be explained by minor diffuse changes in the diffusion tensor imaging metrics inside the tracts. These types of changes are observable by ROI methods, while tract-based spatial statistics may omit minor findings owing to its skeletonising steps. Due to skeletonisation in tract-based spatial statistics, different areas of white matter are effectively studied in tract-based spatial statistics and atlas-based analysis [65]. Our results demonstrate that the atlas-based analysis approach is beneficial when studying children’s diffusion tensor imaging data. In summary, it is likely that tract-based spatial statistics and atlas-based analysis analyse different brain white matter tracts, and although they complement each other, they may lead to differing results.

The small sample size is a limitation of this study. However, the study population belonged to a prospective cohort followed from the fetal period [35] and the control group of children born appropriate for gestational age was gestational age- and gender-matched, minimising the effect of prematurity on the results. Severely injured children from the original cohort of growth restricted fetuses were unable to participate, and none of the participating children had cerebral insults during the perinatal and neonatal periods. Thus, our results effectively reflect the outcome in a group of children born with fetal growth restriction with the most optimal outcome. The children born with fetal growth restriction and their counterparts with normal fetal growth had their MRI scans performed within the same age range, and all participants were imaged on the same MRI scanner using the same sequences. Furthermore, quality control was carried out for diffusion tensor imaging data, excluding the potentially bias-inducing artefact-ridden data from the diffusion tensor imaging analyses.

## Conclusion

Children born with fetal growth restriction at 24–40 weeks had smaller total intracranial volumes than their peers with normal fetal growth, with no difference in grey and white matter volumes. Grey and white matter volumes show no difference between the compared groups, not even in the presence of significant placental insufficiency (UA PI > 2SD). Significant alterations in white matter microstructure in atlas-based analysis in 8- to 10-year-old children born with fetal growth restriction indicate that poor fetal growth has an impact on white matter maturation and may impair later neurodevelopment.

Further data on the impact of fetal circulatory changes in fetal growth restriction, short-term outcome, and postnatal development on cerebral maturation and pathology are needed to optimise the timing of delivery and to develop targeted therapeutic measures to support optimal long-term outcome.
